# Epidemiological and clinical features in patients with coronavirus disease 2019 outside of Wuhan, China: Special focus in asymptomatic patients

**DOI:** 10.1371/journal.pntd.0009248

**Published:** 2021-03-10

**Authors:** Ping Liu, Ruichao Niu, Jie Chen, Yuling Tang, Wenfang Tang, Linyan Xu, Juntao Feng

**Affiliations:** 1 Department of Respiratory Medicine, the First Hospital of Changsha, Changsha, Hunan, China; 2 Department of Respiratory Medicine, Xiangya Hospital, Central South University, Changsha, Hunan, China; King Saud University College of Medicine, SAUDI ARABIA

## Abstract

**Objectives:**

In December 2019, coronavirus disease 2019 (COVID-19) emerged in Wuhan City and rapidly spread across the world. The clinical characteristics of affected patients in different regions and populations may differ. Thus, this study aimed to identify the characteristics of the disease to provide an insight about the prevention and treatment of COVID-19.

**Methods:**

Data on the demographic characteristics and clinical findings of the patients admitted at the First Hospital of Changsha from January 1, 2020 to February 10, 2020 were assessed.

**Results:**

In this study, there were 8 (3.8%) asymptomatic, 21 (10.0%) mild upper respiratory tract infection (URTI), and 180 (86.1%) pneumonia cases. In total, 47 (22.5%) patients resided in Wuhan, and 45 (21.5%) had recently traveled to Wuhan before disease onset. Moreover, 19 (9.1%) had contact with people from Wuhan, and 69 (33.0%) were family cluster cases. The median incubation period was approximately 6.3 (range: 1.0–20.0) days. Fever and cough were the most common initial symptoms: 99 (49.3%) patients presented with fever, without cough; 59 (29.4%) with cough, without fever; and 33 (16.4%) with both fever and cough.

**Conclusion:**

The symptoms of patients with COVID-19 were relatively mild outside Wuhan, and family cluster was a remarkable epidemic characteristic. Special attention should be paid to asymptomatic patients.

## Author summary

We reported the epidemiological, clinical characteristics and laboratory findings of case series of 209 patients with laboratory-confirmed SARS-CoV-2 infection in Changsha, Hunan province. In addition, we compared the characteristics of patients among asymptomatic, mild upper respiratory tract infection and viral pneumonia of SARS-CoV-2 infection in detail. The symptoms of patients with COVID-19 were relatively mild outside Wuhan, and family cluster was a remarkable epidemic characteristic. Special attention should be paid to asymptomatic patients.

## Introduction

In December 2019, a cluster of pneumonia cases of unknown etiology was reported in Wuhan, Hubei Province, China [1]. A novel coronavirus, referred to as the severe acute respiratory syndrome coronavirus 2 (SARS-CoV-2; provisionally named 2019 novel coronavirus [2019-nCoV]), was subsequently detected via a deep sequencing analysis of lower respiratory tract samples [2,3]. Genomic sequences also support the notion that SARS-CoV-2 is closely correlated to bat-derived SARS-like coronaviruses [4]. Recent studies have shown that SARS-CoV-2 has a strong affinity to human respiratory receptors, which indicates that it has a strong capability to infect humans [3].

Although the initial cases of coronavirus disease 2019 (COVID-19) were found to be correlated to Wuhan’s Huanan Seafood Market, the source and possible intermediate animal vectors and the mechanisms underlying the spread of COVID-19 are still unknown [5,6]. Since January 2020, COVID-19 has rapidly spread from Wuhan to the entire country. Since January 18, 2021, 98,794 laboratory-confirmed cases of COVID-19 have been documented in Mainland China [7]. In recent days, COVID-19 has spread in different countries globally, with outbreaks in Spain and Italy, and there is also a significant increase in the number of cases in the United States. By January 18, 2021, 93,805,612 infections and 2,026,093 deaths had been reported worldwide, and the numbers can increase further [7]. Therefore, COVID-19 has become a major public health issue and has been declared as a Public Health Emergency worldwide.

Although numerous studies have been carried out, there is still no effective vaccine or antiviral treatment for COVID-19. The early diagnosis and understanding of the transmission patterns is crucial in fighting the COVID-19 outbreak. Previous studies have shown the epidemiological, clinical, laboratory, and radiological characteristics of patients with COVID-19 [5,8–10]. However, specific data about the characteristics of patients outside Wuhan are limited. In addition, the characteristics of patients with asymptomatic and mild upper respiratory tract infection (URTI) are not fully elucidated.

Herein, we investigate the epidemiological, clinical, and laboratory findings of 209 patients with laboratory-confirmed COVID-19 in Changsha, Hunan Province, with focus on asymptomatic infection, mild URTI, and pneumonia cases. To provide an insight about the prevention and treatment of COVID-19.

## Methods

### Ethics statement

The First Hospital of Changsha, Changsha, Hunan Province, was designated to treat all COVID-19 patients in Changsha. Admissions between January 20, 2020 to February 10, 2020 were included with data followed through March 30, 2020. This retrospective study was approved by the ethics committee of the First Hospital of Changsha (KL-2020005). Written informed consent was obtained from the [individual(s) and/or minor(s)’ legal guardian/next of kin] for the publication of any potentially identifiable images or data included in this article retrospectively.

### Study design

People who met one of the following standards were asked to do the SARS-Cov-2 nucleic acid test in Changsha, Hunan Province, China. First, people had close contact with the confirmed cases of COVID-19; Second, people had a recent travel to the epidemic area (Wuhan); Third, people had close contact with patients who had respiratory symptoms like fever or cough and lived in the same community with confirmed cases of COVID-19; Fourth, people had respiratory symptoms like fever or cough. To exclude other respiratory diseases, the patients who lacked fever but showed only cough at admission were subjected to procedures such as routine blood laboratory analysis, chest CT, and more than three times of SARS-Cov-2 nucleic acid test. Then experienced respiratory medical doctors and radiologists would make the final diagnosis together basing on the results of those tests.

All the patients with positive result to high-throughput sequencing or real-time reverse transcription polymerase chain reaction (RT-PCR) assay using nasal and pharyngeal swab specimens were sent to The First Hospital of Changsha in Changsha, no matter he or she has symptoms or not. For the asymptomatic cases, they were just given concentrate isolation and medical observation for 2 weeks but no treatment on admission. If the asymptomatic cases developed symptoms during the period, they became viral pneumonia cases and were given therapy. If they had no symptoms during the 2 weeks, they would be discharged when two consecutive SARS-Cov-2 nucleic acid tests were negative.

### Definitions

Confirmed COVID-19 case was defined as a positive result to high-throughput sequencing or RT-PCR assay using nasal and pharyngeal swab specimens [5,10]. Asymptomatic case was defined as a confirmed COVID-19 case but without the involvement of any symptoms or radiographic evidence of pneumonia during the follow-up days. Mild URTI was defined as a confirmed COVID-19 case involving fever or any respiratory symptoms but without radiographic evidence of pneumonia. Pneumonia was defined as a confirmed case involving both fever and/or respiratory symptoms with radiographic evidence of pneumonia. The initial symptoms were defined as the symptom observed upon disease onset. The severity of COVID-19 was defined according to the international guidelines for community-acquired pneumonia [11]. The incubation period was defined as the number of days between the estimated exposure and the onset of symptoms. Shock and acute respiratory distress syndrome (ARDS) were defined in accordance with the World Health Organization guidelines for COVID-19 [12]. Acute kidney injury was diagnosed according to serum creatinine levels [13]. Rhabdomyolysis was defined as muscle pain or muscle weakness upon admission, and the creatine kinase level was 10 times higher than the upper normal limit [14].

### Data collection

Two experienced respiratory medical doctors collected data about the clinical characteristics of the participants from the electronic medical records. A standardized case report form was designed to collect clinical data, including demographic data, personal history, exposure history, underlying comorbidities, sign and symptoms, laboratory findings, radiological characteristics, treatment measures, and outcomes. If the information was not clear, we directly communicated with the doctor responsible for the treatment of the patient for clarification. Two researchers independently reviewed the data collection forms to double check the information.

### Statistical analysis

The Statistical Package for the Social Sciences software version 20.0 (Chicago, IL, the USA) was used for statistical analysis. Categorical variables were expressed as frequency counts and percentages with 95% confidence interval (CI). Continuous variables were presented as either means and standard deviations or medians with interquartile ranges. Chi-square test and Fisher’s exact test were used to analyze categorical variables between the groups, and student’s *t*-test, Mann–Whitney U test, and Kruskal–Wallis test were used accordingly to compare continuous variables. A two-tailed *p* value <0.05 was considered statistically significant.

## Results

### Epidemiologic features

**Fig 1:**
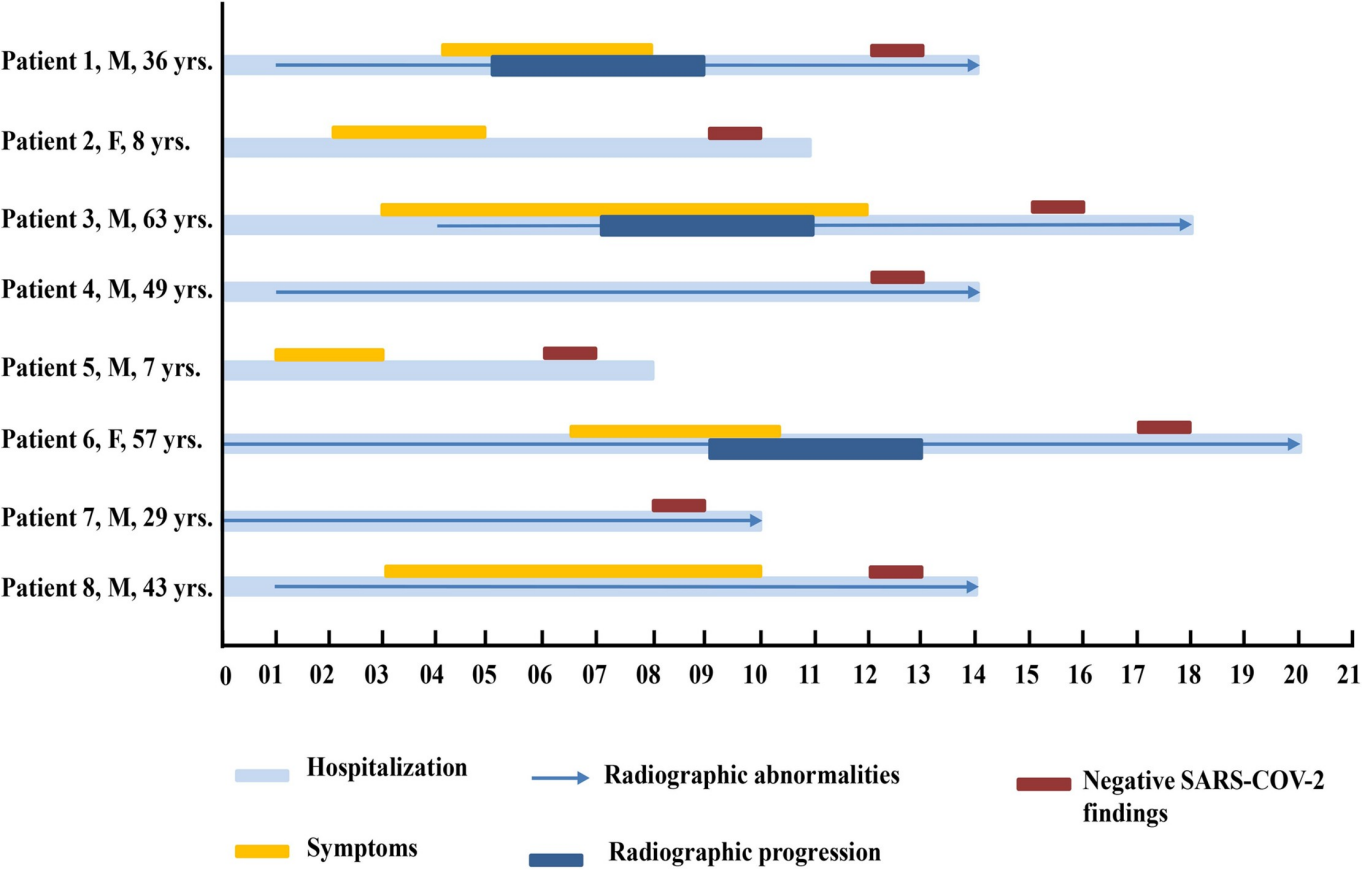
Chronology of symptoms, radiographic findings of the eight COVID-19 patients who developed symptoms or signs during follow-up. The numbers on the bottom indicate the number of days since admission. yrs, years old.

By February 10, 2020, 209 inpatients had laboratory-confirmed COVID-19 in Changsha Hunan Province. Sixteen cases were asymptomatic at admission, among these 8 maintained asymptomatic picture during the hospitalization while the rest became symptomatic at 1–6 days of admission. Of the 8 patients who developed symptoms, 6 showed chest radiographic abnormalities, and 3 of them revealed radiographic progression (Fig 1). Moreover, of the total symptomatic patients, 21 (10.0%) presented with mild URTI and 180 (86.1%) with pneumonia. In terms of age, the median age was 45.2 (range: 1–84) years for all the participants. For the symptomatic patients, the median age in mild URTI and viral pneumonia group were 33.6 (range: 1.0–63.0) and 47.7 (range: 17.0–84.0) years respectively, and both of them were significantly older than that in the asymptomatic infection group (19.8 years, range: 1–53; *P* < 0.001). In terms of age group, for all the participants, 70 (33.5%) patients were aged 30–44 years; 55 (26.3%), 45–59 years; 50 (23.9%), ≥60 years; 27 (12.9%), 14–29 years; and 7 (3.3%), <14 years. For asymptomatic group, 4 out of 8 patients (50%) were aged <14 years, and the proportion was significantly higher than that in symptomatic group patients (*P* < 0.001). For mild URTI group, 12 out of 21 patients (57.1%) were aged 30–44 years, and the proportion was significantly higher than that in asymptomatic group and viral pneumonia group (*P* < 0.001) (Table 1). Regarding sex, for all the participates, 106 out of 209 (50.7%) patients were men. For asymptomatic patients, 3 out of 8 patients were male. For symptomatic patients, 9 out of 21 (42.9%) and 94 out of 180 (52.2%) were male in mild URTI and viral pneumonia, respectively. None of the patients had a history of exposure to Huanan Seafood Market. Of the 209 patients, 47 (22.5%) resided in Wuhan, and 45 (21.5%) had a recent travel to Wuhan before disease onset. Moreover, 19 (9.1%) had contact with people from Wuhan, and 69 (33.0%) were family cluster cases. Family cluster cases were significantly more common in asymptomatic infection group (7/8, 87.5%) than that in mild URTI (12/21, 57.1%) and viral pneumonia group (50/180, 27.8%) (*P* <0.001). In total, 57 (27.3%) patients could provide the exact date of the close contact with individuals with confirmed or suspected COVID-19. The median incubation period was approximately 6.3 (range: 1–20) days based on the interval between the date of symptom onset and first exposure (Table 1).

**Table 1:** Clinical characteristics of COVID-19 patients stratified by categories based on symptoms severity.

Variable	All patients N = 209 (100%)	Asymptomatic infection N = 8 (3.8%)	Mild URTI N = 21 (10.0%)	Viral pneumonia N = 180 (86.1%)	*P* value^&^
**Age, Median (range)—yrs**	45.2 (1.0–84.0)	19.8 (1.0–53.0)**	33.6 (1.0–63.0)	47.7 (17.0–84.0)	<0.001
**Age groups—No., %**					
** **< 14 yrs	7 (3.3)	4 (50)** ^#^	3 (14.3)**	0 (0)	<0.001
** **14–29 yrs	27 (12.9)	3 (37.5)	3 (14.3)	21 (11.7)	0.101
** **30–44 yrs	70 (33.5)	0 (0) ^##^	12 (57.1)*	58 (32.2)	<0.01
** **45–59 yrs	55 (26.3)	1 (12.5)	1 (4.8)*	53 (29.4)	0.035
** **≥ 60 yrs	50 (23.9)	0 (0)	2 (9.5)	48 (26.7)	0.059
**Male—No., %**	106 (50.7)	3 (37.5)	9 (42.9)	94 (52.2)	0.538
**Exposure to source of transmission within 14 days–No.,%**					
** **Come from Wuhan	47 (22.5)	1 (12.5)	4 (19.0)	42 (23.3)	0.714
** **Recently been to Wuhan	45 (21.5)	2 (25.0)	4 (19.0)	39 (21.7)	0.934
** **Contacted with people from Wuhan	19 (9.1)	0 (0)	3 (14.3)	16 (8.9)	0.474
** **Contacted with confirmed cases	57 (27.3)	4 (50.0)	8 (38.1)	45 (25.0)	0.150
No exposure history	32 (15.3)	0 (0)	1 (4.8)	31 (17.2)	0.153
**Familial cluster–No.,%**	69 (33.0)	7 (87.5) **	12 (57.1)**	50 (27.8)	<0.001
**Incubation period, Median (range)—days**	6.3 (1.0–20.0)	-	5.8 (3.0–9.0)	6.3 (1.0–20.0)	0.685
**Smoking history–No.,%**					
Never smokers	180 (86.1)	8 (100.0)	18 (85.7)	154 (85.6)	0.512
Ex-smokers	8 (3.8)	0 (0)	1 (4.8)	7 (3.9)	0.831
Current smokers	21 (10.0)	0 (0)	2 (9.5)	19 (10.6)	0.621
**Coexisting conditions–No.,%**					
** **Total number of coexisting conditions	73 (34.9)	1 (12.5)	3 (14.3)*	69 (38.3)	0.036
** **Hypertension	29 (13.9)	0 (0)	2 (9.5)	27 (15.0)	0.404
** **Diabetes	10 (4.8)	0 (0)	0 (0)	10 (5.6)	0.429
** **Chronic obstructive pulmonary diseases	5 (2.4)	0 (0)	0 (0)	5 (2.8)	0.662
** **Coronary heart disease	4 (1.9)	0 (0)	0 (0)	4 (2.2)	0.720
** **Cerebrovascular diseases	3 (1.4)	0 (0)	0 (0)	3 (1.7)	0.783
** **Renal disease	1 (0.5)	0 (0)	0 (0)	1 (0.6)	0.922
** **Hepatobiliary disease	11 (5.3)	0 (0)	0 (0)	11 (6.3)	0.392
** **Cancers	1 (0.5)	0 (0)	0 (0)	1 (0.6)	0.922
** **Hematological tumors	1 (0.5)	0 (0)	0 (0)	1 (0.6)	0.922
** **Graves disease	3 (1.4)	0 (0)	0 (0)	3 (1.7)	0.783
** **Rheumatic connective tissue disease	2 (1.0)	0 (0)	0 (0)	2 (1.1)	0.850
** **Congenital heart disease	3 (1.4)	1 (12.5) **	1 (4.8)	1 (0.6)	<0.01
**Initial symptoms–No.,%**					
Fever without cough	99 (49.3)	0 (0)	14 (66.7)	85 (47.2)	0.092
Days from fever to cough, Median (range)—days	3.4 (1.0–7.0)	-	3.2 (1.0–5.0)	3.6 (1.0–7.0)	0.596
** **Cough without fever	59 (29.4)	0 (0)	4 (19.0)	55 (30.6)	0.273
** **Days from cough to fever, Median (range)–days	3.2 (1.0–9.0)	-	3.0 (1.0–7.0)	3.3 (1.0–9.0)	0.531
** **Both fever and cough	33 (16.4)	0 (0)	0 (0)*	33 (18.3)	0.043
** **Others	10 (5.0)	0 (0)	3 (14.3)*	7 (3.9)	0.087
**Signs and symptoms—No.,%**					
Fever on admission	62 (29.7)	0 (0)	4 (19.0)	58 (32.2)	0.079
** **Temperature on admission, °C					
** <** 37.3	147 (70.3)	8 (100.0)	17 (81.0)	122 (67.8)	0.079
** **37.3–38.0	48 (23.0)	0 (0)	4 (19.0)	44 (24.4)	0.248
** **38.1–39.0	14 (6.7)	0 (0)	0 (0)	14 (7.8)	0.299
** >** 39.0	0 (0)	0 (0)	0 (0)	0 (0)	-
Fever during hospital admission	155 (74.2)	0 (0) ** ^##^	17 (81.0)	138 (76.7)	<0.001
** **Highest temperature during hospital admission, °C					
** <** 37.3	54 (25.8)	8 (100.0) ** ^##^	4 (19.0)	42 (23.3)	<0.001
** **37.3–38.0	72 (34.4)	0 (0)	12 (57.1)*	60 (33.3)	0.011
** **38.1–39.0	65 (31.1)	0 (0)	3 (14.3)*	62 (34.4)	0.026
** >** 39.0	25 (12.0)	0 (0)	2 (9.5)	23 (12.8)	0.517
Cough	130 (64.7)	0 (0)	8 (38.1)**	122 (67.8)	<0.001
Sputum production	64 (31.8)	0 (0)	1 (4.8)**	63 (35.0)	<0.001
** **Dyspnea	27 (31.8)	0 (0)	0 (0)	27 (15.0)	0.056
** **Hemoptysis	1 (0.50)	0 (0)	0 (0)	1 (0.60)	0.732
** **Sore throat	20 (10.0)	0 (0)	5 (23.8)*	15 (8.3)	0.025
** **Diarrhea	6 (3.0)	0 (0)	1 (4.8)	5 (2.8)	0.613
** **Nausea or vomiting	3 (1.5)	0 (0)	0 (0)	3 (1.7)	0.551
** **Fatigue	34 (16.9)	0 (0)	5 (23.8)	29 (16.1)	0.373
** **Myalgia or arthralgia	17 (8.5)	0 (0)	1 (4.8)	16 (8.9)	0.520
** **Headache	6 (3.0)	0 (0)	2 (9.5)	4 (2.2)	0.063
** **Chill	17 (8.5)	0 (0)	1 (4.8)	16 (8.9)	0.520

URTI: upper respiratory tract infection

^&^ Indicate results of comparison of asymptomatic infection versus mild URTI versus viral pneumonia

** Compared to viral pneumonia group *P* < 0.01

* Compared to viral pneumonia group *P* < 0.05

^##^ Compared to URTI group *P* < 0.01

^#^ Compared to URTI group *P* < 0.05

### Clinical features

Fever (74.2%) and cough (64.7%) were the most common symptoms of COVID-19, and diarrhea (3.0%) and nausea or vomiting (1.5%) were rare. Regarding the initial symptoms, 99 (49.3%) patients presented with fever but without cough, 59 (29.4%) with cough but without fever, and 33 (16.4%) with both fever and cough. The median duration from fever to cough was 3.4 (range: 1.0–7.0) days, and that from cough to fever was 3.2 (range: 1.0–9.0) days (Table 1). Less than half of the patients had at least one underlying comorbidity, such as hypertension (29/209, 13.9%), diabetes (10/209, 4.8%), hepatobiliary disease (11/209, 5.3%), and chronic obstructive pulmonary disease (COPD) (5/209, 2.4%) (Table 1). The total number of underlying comorbidities in mild URTI group (3/21, 14.3%) was much smaller than that in viral pneumonia group (69/180, 38.3%) (*P* < 0.05).

Regarding laboratory findings, there were no abnormities in the group of asymptomatic infection. Leucopenia (white blood cell count < 4×10^9^/L) and lymphopenia (< 1.0 ×10^9^/L) was observed in 15 (7.2%) and 33 (15.8%) of 209 patients, respectively, upon admission. Increased white blood cell count (30.6% vs. 0%) and neutrophil count (29.4% vs. 0%) and decreased lymphocyte count (18.3% vs. 0%) were relatively more common in viral pneumonia group than that in mild URTI group (*P* < 0.05). In addition, increased C-reactive protein (66.1% vs. 14.3%, *P* < 0.001) and erythrocyte sedimentation rate (82.2% vs. 14.3%, *P* < 0.001) were also more common in viral pneumonia cases than in mild URTI cases.

**Fig 2:**
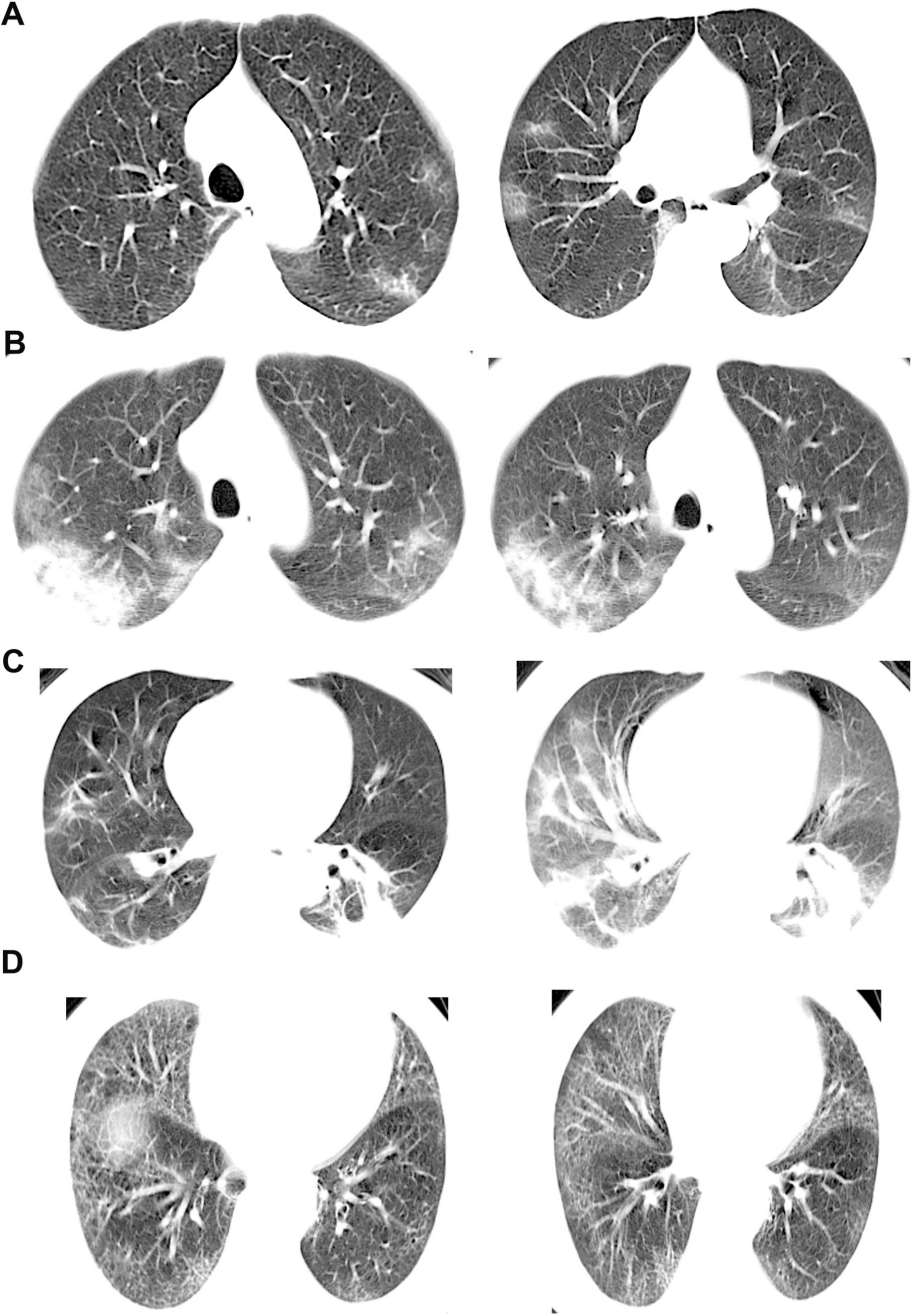
Characteristics of chest computed tomography scan in COVID-19 patients. A) A 51-year-old woman with a history of contact with a patient with confirmed who presented with fever and cough for 2 days. Non-contrast enhanced chest computed (CT) scan revealed multiple ground glass opacification in the left upper lobe and the middle lobe. B) A 46-year-old man with a recent history travel to Wuhan presented with cough for 9 days, fatigue for 7 days and fever for 4 days. Non-contrast-enhanced chest CT scan revealed patchy/punctate ground glass opacification in both upper lobes. C) A 65-year-old man with a recent history of travel to Wuhan presented with fever and cough for 2 days. Non-contrast-enhanced chest CT scan revealed patchy consolidation in both the lower lobes. D) A 66-year-old man with a history of contacting with confirmed COVID-19 presented with fever for 3 days. Non-contrast-enhanced chest CT scan revealed interstitial abnormalities in both lung fields.

Chest computed tomography scan revealed ground glass opacity (n = 56, 26.8%), patchy/punctate ground glass opacities (n = 107, 51.2%), patchy consolidation (n = 34, 16.3%), and interstitial abnormalities (n = 19, 9.1%) at presentation (Table 2). The typical chest computed tomography scan findings are shown in Fig 2.

**Table 2:** Laboratory and chest radiography findings of COVID-19 patients stratified by categories based on symptoms severity.

Laboratory and chest radiography findings	All patientsN = 209 (100%)	Asymptomatic infection N = 8 (3.8%)	Mild URTI N = 21 (10.0%)	Viral pneumonia N = 180 (86.1%)	*P* value^&^
**Laboratory findings-No.,%**					
White blood cell count					
** **Decreased	15 (7.2)	0 (0)	0 (0)	15 (8.3)	0.272
Normal	139 (66.5)	8 (100)	21 (100)**	110 (61.1)	<0.001
** **Increased	55 (26.3)	0 (0)	0 (0)*	55 (30.6)	0.002
Neutrophil count					
** **Decreased	18 (8.6)	0 (0)	0 (0)	18 (10.0)	0.205
Normal	138 (66.0)	8 (100)	21 (100)**	109 (60.6)	<0.001
** **Increased	53 (25.4)	0 (0)	0 (0)*	53 (29.4)	0.003
Lymphocyte count					
** **Decreased	33 (15.8)	0 (0)	0 (0)*	33 (18.3)	0.043
** **Normal	173 (83.3)	8 (100)	21(100)*	145 (80.6)	0.034
** **Increased	2 (1.0)	0 (0)	0 (0)	2 (1.1)	0.850
Platelet count					
** **Decreased	8 (3.8)	0 (0)	0 (0)	8 (4.4)	0.512
** **Normal	196 (93.8)	8 (100)	21 (100)	167 (92.8)	0.327
** **Increased	5 (2.4)	0 (0)	0 (0)	5 (2.8)	0.662
Hemoglobin					
** **Decreased	11 (4.3)	0 (0)	1 (4.8)	10 (5.6)	0.784
** **Normal	198 (94.7)	8 (100)	20 (95.2)	170 (94.4)	
Alanine aminotransferase					
** **Normal	189 (90.4)	8 (100)	20 (95.2)	161 (89.4)	0.447
** **Increased	20 (9.6)	0 (0)	1 (4.8)	19 (10.6)	
Aspartate aminotransferase					
** **Normal	185 (88.5)	8 (100)	20 (95.2)	157 (87.2)	0.322
** **Increased	24 (11.5)	0 (0)	1 (4.8)	23 (12.8)	
Total bilirubin					
** **Normal	179 (85.6)	8 (100)	21 (100)	150 (83.3)	0.060
** **Increased	30 (14.4)	0 (0)	0 (0)	30 (16.7)	
Creatinine					
** **Normal	207 (99.0)	8 (100)	21 (100)	178 (98.9)	0.850
** **Increased	2 (1.0)	0 (0)	0 (0)	2 (1.1)	
Creatine kinase					
** **Normal	192 (91.9)	8 (100)	20 (95.2)	164 (91.1)	0.558
** **Increased	17 (8.1)	0 (0)	1 (4.8)	16 (8.9)	
Lactate dehydrogenase					
** **Normal	179 (85.6)	8 (100)	19 (90.5)	152 (84.4)	0.377
** **Increased	30 (14.4)	0 (0)	2 (9.5)	28 (15.6)	
D-dimer					
** **Normal	162 (77.5)	8 (100)	21 (100)*	133 (73.9)	0.008
** **Increased	47 (22.5)	0 (0)	0 (0)	47 (26.1)	
C-reactive protein level					
** **Normal	87 (41.6)	8 (100)	18 (85.7)**	61 (33.9)	<0.001
** **Increased	122 (58.4)	0 (0)	3 (14.3)	119 (66.1)	
Erythrocyte sedimentation rate					
** **Normal	58 (27.8)	8 (100)	18 (85.7)**	32 (17.8)	<0.001
** **Increased	151 (72.2)	0 (0)	3 (14.3)	148 (82.2)	
Procalcitonin					
** **Normal	200 (95.7)	8 (100)	21 (100)	171 (95.0)	0.469
** **Increased	9 (4.3)	0 (0)	0 (0)	9 (5.0)	
Sodium					
** **Decreased	12 (5.7)	0 (0)	1 (4.8)	11 (6.1)	0.752
Normal	192 (91.9)	8 (100)	20 (95.2)	164 (91.1)	0.558
** **Increased	5 (2.4)	0 (0)	0 (0)	5 (2.8)	0.662
Potassium					
** **Decreased	9 (4.3)	0 (0)	1 (4.8)	8 (4.4)	0.827
** **Normal	198 (94.7)	8 (100)	20 (95.2)	170 (94.4)	0.784
** **Increased	2 (1.0)	0 (0)	0 (0)	2 (1.1)	0.850
Chloride					
** **Decreased	7 (3.3)	0 (0)	0 (0)	7 (3.9)	0.558
** **Normal	200 (95.7)	8 (100)	21 (100)	171 (95.0)	0.469
** **Increased	2 (1.0)	0 (0)	0 (0)	2 (1.1)	0.850
**Chest radiography findings- No.,%**					
Ground-glass opacity	56 (26.8)	0 (0)	0 (0)*	56 (31.1)	0.002
Patchy/ punctate ground glass opacities	107 (51.2)	0 (0)	0 (0)**	107 (59.4)	<0.001
Patchy consolidation	34 (16.3)	0 (0)	0 (0)*	34 (18.9)	0.038
Interstitial abnormalities	19 (9.1)	0 (0)	0 (0)	19 (10.6)	0.186

URTI: upper respiratory tract infection

^&^ Indicate results of comparison of mild URTI versus viral pneumonia

** Compared to viral pneumonia group P < 0.01

* Compared to viral pneumonia group P < 0.05

### Clinical outcomes

Seventeen cases in the viral pneumonia group were severity cases (17/180, 9.4%). In patients admitted in the hospital, the common complications was pneumonia (23.3%), followed by rhabdomyolysis (8.9%), ARDS (3.9%), septic shock (1.1%), disseminated intravascular coagulation (1.1%), and acute kidney injury (0.6%) in the group of viral pneumonia. (Table 3).

**Table 3:** Complications, treatment and outcomes of COVID-19 patients stratified by categories based on symptoms severity.

Characteristics	All patients N = 209 (100%)	Asymptomatic infection N = 8 (3.8%)	Mild URTI N = 21 (10.0%)	Viral pneumonia N = 180 (86.1%)	*P* value
**Severity cases–No., %**	17 (8.1)	0 (0)	0 (0)	17 (9.4)	0.225
**Complications–No., %**					
Pneumonia	42 (20.1)	0 (0)	0 (0)*	42 (23.3)	0.014
ARDS	7 (3.3)	0 (0)	0 (0)	7 (3.9)	0.558
Septic shock	2 (1.0)	0 (0)	0 (0)	2 (1.1)	0.850
AKI	1 (0.5)	0 (0)	0 (0)	1 (0.6)	0.922
DIC	2 (1.0)	0 (0)	0 (0)	2 (1.1)	0.850
Rhabdomyolysis	16 (7.7)	0 (0)	0 (0)	16 (8.9)	0.248
**Treatments–No., %**					
Antiviral therapy	201 (96.2)	0 (0)	21 (100.0)**	180 (100.0)	<0.001
Antibiotic therapy	70 (33.5)	0 (0)	1 (4.8)*	69 (38.3)	0.001
Use of corticosteroid	27 (12.9)	0 (0)	0 (0)	27 (15.0)	0.082
CRRT	1 (0.5)	0 (0)	0 (0)	1 (0.6)	0.922
**Oxygen therapy**					
Nasal cannula	48 (23.0)	0 (0)	0 (0)**	48 (26.7)	0.007
High-flow nasal cannula	9 (4.3)	0 (0)	0 (0)	9 (5.0)	0.469
Non-invasive ventilation	5 (2.4)	0 (0)	0 (0)	5 (2.8)	0.662
Invasive mechanical ventilation	3 (1.4)	0 (0)	0 (0)	3 (1.7)	0.783
**Prognosis–No., %**					
Discharge	207 (96.2)	8 (100)	21 (100)	172 (95.6)	0.512
Death	2 (1.0)	0 (0)	0 (0)	2 (1.1)	0.850

URTI: upper respiratory tract infection; ARDS: acute respiratory distress syndrome; AKI: acute kidney injury; DIC: disseminated intravascular coagulation; CRRT: continuous renal replacement therapy.

** Compared to viral pneumonia group P < 0.01

* Compared to viral pneumonia group P < 0.05

Both mild URTI and viral pneumonia patients received antiviral therapy (include interferon α, lopinavir, ritonavir and ribavirin), and 69 (33.5%) were managed with empirical antibiotic therapy. Moreover, corticosteroid was administered in 27 (12.9%) patients, and one (0.5%) received continuous renal replacement therapy. In addition, high-flow nasal cannula therapy, non-invasive ventilation, and invasive mechanical ventilation were initiated in 5.0%, 2.8%, and 1.7% of viral pneumonia patients, respectively (Table 3).

In total, 207 (96.2%) patients had been discharged, and two (1.0%) died. Both patients (aged 58 and 64 years) who died presented with severe symptoms (Table 3).

## Discussion

This retrospective case series assessed the clinical characteristics of 209 patients with COVID-19 outside of Wuhan, China, with a focus on epidemiologic features and clinical course. The differences among patients with asymptomatic infection, mild URTI and viral pneumonia were analyzed.

Understanding the epidemiological history is essential in controlling the COVID-19 outbreak. In this study, 32 (15.3%) of 209 patients did not have any definite exposure history. Of these patients, six developed severe pneumonia. This phenomenon can be explained by the undetected pool of covert cases showing limited to no symptoms, which could be a potential source of infection [15]. Therefore, evaluating the proportion of asymptomatic cases is critical for the assessment of the epidemic potential of COVID-19.

Based on our data, the prevalence rates of asymptomatic infection, mild URTI, and pneumonia were 3.8%, 13.9%, and 86.1%, respectively. These results were consistent with those of other studies, which showed that asymptomatic cases accounted for 5% of all confirmed cases in Beijing [16]. However, a report about the cases in the Diamond Princess showed that asymptomatic cases accounted for 18% of all the infected cases (n = 700) [17]. The percentage of asymptomatic cases was also reported to be 31% of all the 565 Japanese people who were evacuated from Wuhan [18]. Those inconsistence findings might be attributed to the insufficient nucleic acid testing performed during the preclinical examinations. The differences in the sample size might have also affected the results. Future serological investigations are needed to determine the actual proportion of asymptomatic cases. In addition, we found that young patients (<14 years) were at risk of asymptomatic or mild COVID-19, which was similar to the report of Hu et al [19]. Notably, in this study, most asymptomatic patients or those with mild symptoms had a clear exposure history and belonged to the familial cluster. These findings indicate that isolation and protective measures, such as wearing of masks, single room isolation, and cancelling of family gatherings, should be strengthened for close contacts, particularly among young individuals.

Although 16 patients were asymptomatic on admission, 8 of them are asymptomatic throughout the course of the disease, the remaining of them developed symptoms or revealed radiographic abnormalities during follow-up. It indicates that asymptomatic period sometimes maybe just a part of the natural course of COVID-19 [20]. For the patients 1–5, 8, they admitted without any subjective symptoms or signs but developed later, so the asymptomatic period is just their incubation period. For the patient 6 and 7, they admitted with radiographic abnormalities but without any symptoms. The asymptomatic period is in their period of illness. All the asymptomatic patients may have the potential to infect others. Recent studies showed that the viral load in the samples obtained from asymptomatic individuals was similar to that obtained from symptomatic individuals [21,22], and this finding provides evidence of the transmission potential of asymptomatic patients [23]. Therefore, to prevent transmission of COVID-19, nucleic acid testing must be performed to identify asymptomatic patients or those with mild symptoms. Future studies are required to find the mode of contagion of asymptomatic cases to develop more control strategies.

Based on the results of recent reports [10,24], fever and cough were the most common initial symptoms of COVID-19. However, fever was only observed in 49.3% of patients upon the initial presentation, and it developed in 74.2% of patients after hospitalization. In total, 59 (29.4%) of 209 presented with cough but without fever. Fever occurred after cough at an average of 3.2 days in some patients whose initial symptoms were only cough. If we only focus on temperature assessment, such patients may be easily missed, and they become the potential source of infection. Therefore, patients with fever, cough, and other upper respiratory tract symptoms should be encouraged to go to the hospital at an early time and undergo nucleic acid testing. Doing massive testing may increase the medical economic burden but it is worth doing due to the great communicability of SARS-CoV-2. Economic evaluation studies of comparison of cost effectiveness of massive testing *versus* the using COVID-19 testing basing on symptoms should be recommended.

In our report, patients in viral pneumonia group had more underlying diseases than that in asymptomatic or mild URTI group. Moreover, viral pneumonia patients presented with a higher white blood cell count and neutrophil count, but with a lower leukocyte count when compared to mild URTI patients. In addition, viral pneumonia patients were more likely to require oxygen therapy and receive antibiotics than mild URTI patients. These findings were not consistent with those of the latest publications [25].

Regarding the therapy for COVID-19, to date, whether there is an effective antiviral drug remains unknown. Less than half (33.5%) of the patients in our study received antibiotics, and corticosteroid was administered in 12.9% of these patients. However, whether the use of antibiotics or steroids affects the prognosis of COVID-19 has not been clearly elucidated. Thus far, meticulous supportive care might have been the only beneficial treatment. Therefore, a vaccine for COVID-19 must be urgently developed to prevent COVID-19 epidemic.

Our study had several limitations. First, due to the retrospective nature of the study, data about the kinetics of viral load and its correlation with clinical progress and transmissibility were not obtained. Second, some cases had missing information about exposure history, symptoms, and laboratory examination findings. Therefore, selection bias might have existed because of the missing information. Third, we only included patients with laboratory-confirmed infection in Changsha during the study period. The number of asymptomatic cases was relatively lower in our study. Some asymptomatic patients or those with mild symptoms might have been hiding in the community, but most of them should be captured in our study due to the massive testing of SARS-Cov-2 nucleic acid.

## Conclusions

The symptoms of patients with COVID-19 in Changsha were relatively mild compared with those in Wuhan. Family cluster is a remarkable epidemic characteristic. Thus, special attention should be paid to asymptomatic patients to prevent the spread of COVID-19. Currently, there are no available drug or vaccine. Thus, further studies must be conducted to develop a high-sensitivity rapid diagnostic reagent and to explore a new effective therapy for COVID-19.
